# Differences in EMG Burst Patterns During Grasping Dexterity Tests and Activities of Daily Living

**DOI:** 10.3389/fbioe.2018.00068

**Published:** 2018-05-25

**Authors:** Jen Rowson, Alaster Yoxall, Victor Gonzalez

**Affiliations:** ^1^Insigneo Institute for In Silico Medicine, Mechanical Engineering, University of Sheffield, Sheffield, United Kingdom; ^2^Art and Design Research Centre, Sheffield Hallam University, Sheffield, United Kingdom; ^3^Department of Musicology, Centre for Interdisciplinary Studies in Rhythm, Time and Motion, University of Oslo, Oslo, Norway

**Keywords:** dexterity, EMG activity, grasping kinematics, activities of daily living, dexterity tests

## Abstract

The aim of this study was to characterize the muscle activation patterns which underlie the performance of two commonly used grasping patterns and compare the characteristics of such patterns during dexterity tests and activities of daily living. EMG of flexor digitorum and extensor digitorum were monitored from 6 healthy participants as they performed three tasks related to activities of daily living (picking up a coin, drinking from a cup, feeding with a spoon) and three dexterity tests (Variable Dexterity Test-Precision, Variable Dexterity Test-Cylinder, Purdue Pegboard Test). A ten-camera motion capture system was used to simultaneously acquire kinematics of index and middle fingers. Spatiotemporal aspects of the EMG signals were analyzed and compared to metacarpophalangeal joint angle of index and middle fingers. The work has shown that a common rehabilitation test such as the Purdue Pegboard test is a poor representation of the muscle activation patterns for activities of daily living. EMG and joint angle patterns from the Variable Dexterity Tests which has been designed to more accurately reflect a range of ADl's were consistently comparable with tasks requiring precision and cylinder grip, reaffirming the importance of object size and shape when attempting to accurately assess hand function.

## Introduction

Two of the biggest challenges in the health care environment are the effectiveness and time-efficiency of treatment. Both factors can be greatly improved by coupling clinical judgment with appropriate and accurate measurement tools. A robust evaluation of patients with hand impairment conditions must include looking at the patient's performance areas within the context of his or her daily living. In a clinic environment, therapists often evaluate common hand function parameters, such as strength, sensibility, and range of motion, along with the administration of dexterity tests, but may forgo to relate assessment procedures with daily living tasks (Aaron and Jansen, 1992; Metcalf et al., 2008; Osu et al., 2011; Gonzalez, 2016).

Hand assessment methods are also tools that can be used for the identification of grip styles. The distinct functional positions of the hand are vital to the evaluation by ensuring assessment of the complete range of grasping patterns. Although there is little conformity to specific classifications of grip styles, they are consistently characterized as: tripod, precision, lateral precision, power, spherical, and extension grip styles (TAYLOR and SCHWARZ, 1955; NAPIER, 1956; Landsmeer, 1962; Gonzalez, 2016).

Dexterity tests are often based upon ordinal scales and are still preferred and widely used in rehabilitation and therapy(Tiffin and Asher, 1948; Fleishman and Hempel, 1954; Peterson and Centre, 1999; Surrey et al., 2003). The main limitations of traditional dexterity tests are low reliability and sensitivity and, more importantly, these tests are not robust enough to correlate well with the hand's wide range of movement and coordination patterns(Aaron and Jansen, 1992; Light et al., 2002; Gonzalez et al., 2015).

Although many hand assessment methods have been designed and implemented, there is little or no uniformity among them, leading to a lack of conformity to a standard test of hand function. Traditionally, measurement of hand function has been time-based and subjective to the assessor's opinion, with tests such as the Purdue Pegboard Test (Tiffin and Asher, 1948), Minnesota Manual Dexterity Test (Surrey et al., 2003), Functional Dexterity Test (Aaron and Jansen, 1992), and Southampton Hand Assessment Procedure (Light et al., 2002) being widely used for rehabilitation and therapy purposes, however, although time is an easy parameter to measure and manipulate statistically, it is not the most accurate and robust measure of hand function (Gonzalez, 2016).

The role of muscle activation patterns and interdependencies in the proficient performance of tasks has not been explored and it is not part of most traditional assessments. This muscle activation can be measured using surface electromyography (SEMG) with signals from SEMG recording muscle tension. SEMG has previously been used for assessment, treatment planning, evaluation of progress and outcomes, rehabilitation, worksite ergonomic design, and research (Duque et al., 1995; Maier and Hepp-Reymond, 1995; Latash et al., 2002; Braido and Zhang, 2004; Castellini and Van Der Smagt, 2013; Berger and d'Avella, 2014).

Previous studies have shown that a hand function assessment method will not provide effective and accurate results unless it is integrated with a robust kinematic and muscle activation signals analysis (Huang et al., 2006; van den Doel et al., 2008). It has also been proved that loss of dexterity implies impairment of the motor coordination patterns required to proficiently perform daily living tasks (Duque et al., 1995; Canning et al., 2000; Liarokapis et al., 2012), however, little is known about the contribution of muscle activation patterns and finger kinematics to the performance of traditional dexterity tests and their relation with activities of daily living.

Computerized three-dimensional kinematic analysis is being increasingly used in clinical practice as a standard tool for the evaluation of interventions in patients with motor or postural dysfunction, especially in the case of gait and spinal posture (Cappozzo et al., 1995, 2005; et al., 2004; Gonzalez, 2016). In the case of the hand, different techniques have been used in the past to analyse motor function, such as goniometers, instrumented gloves or motion tracking from digital images (Ellis and Bruton, 2002; Winges et al., 2003; Dipietro et al., 2008). Many of these techniques do not allow for the simultaneous measurement of all degrees of freedom and may interfere with the normal development of the hand activities. In this sense, the motion tracking of passive markers from video images (motion capture) is a good choice, as although some movement restriction can be introduced by using passive markers, it is much lower than using instrumented gloves or electronic goniometers (Small et al., 1992; Bodenheimer, 1997; Rash et al., 1999; Moeslund and Granum, 2001; Chiari et al., 2005; Degeorges et al., 2005).

Recent advances in motion capture systems coupled to more efficient capture volumes and higher resolution make possible the measurement of representative hand activities, while at the same time reducing the patient's inconvenience and the invasiveness of the tests (Braido and Zhang, 2004; Carpinella et al., 2006; Warlow and Lawson, 2012; Sancho-Bru et al., 2014; Gonzalez, 2016). Simultaneous correlated motion at multiple joints has been studied during more sophisticated uses of the hand, such as typing (Soechting and Flanders, 1997), playing the piano (Engel et al., 1997), or haptic interactions (Thakur et al., 2008). Even when normal subjects are instructed to move one finger, correlated movement occurs in the adjacent fingers (Häger-Ross and Schieber, 2000). In many of these studies of hand movements, principal component analysis (PCA) and correlation coefficients have been applied to show the role of particular coordination patterns in hand function, however, a comparison between finger interdependencies during daily tasks and dexterity tests has yet to be made. Hence, this study uses motion-capture and SEMG to investigate the patterns of muscle activity and finger joint flexion angles involved in commonly used grasping patterns among tasks related to activities of daily living and dexterity tests.

## Aims

This study aims at the quantitative examination of finger coordination from joint angle patterns and muscle activity obtained from motion capture and surface EMG respectively, during the performance of dexterity tests and tasks related to activities of daily living. The analysis presented in this article will help understanding and facilitate comparison of finger movement and coordination patterns from dexterity tests with those from activities of daily living. Hence, the study will provide data to further validate such tests and their suitability as hand function assessment methods and hence aid the development of new tests that better reflect the needs of patients and healthcare practitioners. The study will also provide insight on the role of motor coordination in the performance of dexterous tasks.

## Methodology

### Experimental protocol

This study examined 6 healthy participants (3 male, 3 female, all right-handed, age 22–38 years, 26 ± 6.2 years) performing three separate experiments, the Variable Dexterity Test (Gonzalez et al., 2015), the Purdue Pegboard Test, and tasks related to activities of daily living: picking up a coin, drinking from a glass, and feeding with a spoon. The activities of daily living were selected as representative of tasks requiring the performance of the precision and cylinder grasping patterns. The sample size is underpowered to fully test the reliability and validity of the protocol but will be sufficient to consider feasibility issues and will offer trend level data to indicate the preliminary value of this approach to hand movement measurement (Gonzalez, 2016).

**Figure 1:**
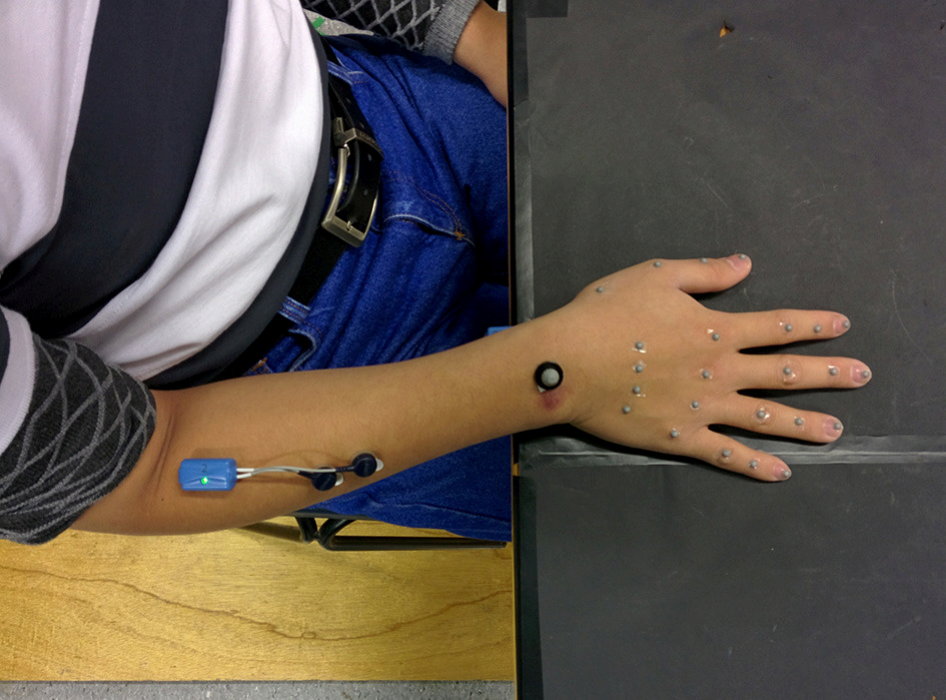
Experimental setup. Subject wearing reflective markers and EMG electrodes.

All movements began in a consistent seated posture with the torso upright, the right upper arm approximately vertical and forearm horizontal, the fingers in natural full extension (abduction/adduction not specified), and the palm resting on a specified area on the table (Figure 1). The participants carried out three repetitions of each experiment with a 10-s pause between each trial (Gonzalez, 2016).

In the first experiment, participants performed two sub-tests of the Variable Dexterity Test (VDT). Both sub-tests required the participant to reach forward over a distance of approximately 25 cm to grasp one object at a time and place it into a hole on a board as rapidly as possible with the right hand. The VDT-Precision sub-test requires the manipulation of a solid object with a rectangular-shaped handle (20 mm tall, 40 mm long, 15 mm thick) using the precision grasping pattern. The VDT-Cylinder sub-test requires the participant to grasp and manipulate a cylinder-shaped handle (80 mm tall, 50 mm diameter). In the second experiment, subjects performed the Purdue Pegboard Test, a standard test used for rehabilitation purposes. In this test the participant reaches forward over a distance of approximately 35 cm to grasp a metal peg (2 mm in diameter), placing it into a hole on the Purdue Pegboard, and returning the hand to the initial posture (Gonzalez, 2016).

In experiment 3, participants performed tasks related to activities of daily living. The tasks for experiment three were selected as activities that require the performance of grasping patterns that could be assessed by both the Variable Dexterity Test and the Purdue Pegboard Test, such as picking up a coin (precision grip), drinking from a glass (cylinder grip), and feeding with a spoon (precision grip). The subjects maintained the same initial posture as in the first experiment and reached forward over a distance of approximately 25 cm to grasp the object (one pound coin, 250 ml glass, and table spoon), performed the task, and placed the object on a specified mark on the table, returning the hand to the initial posture (Gonzalez, 2016).

### Ethical approval for the study

The experimental protocol was approved by the Department of Mechanical Engineering Ethics Committee at the University of Sheffield.

### Data acquisition

The acquisition technique consisted of the placement of 25 reflective markers (24–4 mm markers 4 mm, and 1–8 mm) on different anatomical hand landmarks. From the index to little fingers, five markers were placed as follows: first marker on the metacarpal base, second marker on the knuckle, third on the proximal interphalangeal (PIP) joint, fourth on the distal interphalangeal (DIP) joint and, finally, the fifth marker on the nail. For the thumb, the first marker was placed on the metacarpal base, a second marker on the MCP joint, the fourth on the IP joint and the fifth marker on the nail. One marker was placed on the wrist, aligned with the middle finger, on the wrist dorsum (Gonzalez, 2016).

In order to capture the marker movement a ten-camera Vicon T-160 opto-electronic motion capture system (Oxford Metrics Ltd., UK) recorded the reflective marker movements at a sampling frequency of 120 Hz, and then output the time-varying marker coordinates in a three-dimensional laboratory coordinate system (X–Y–Z) established through calibration (Gonzalez, 2016).

To facilitate kinematic descriptions and to calculate joint angles a local coordinate system X_0_-Y_0_-Z_0_ was established. The origin of this local coordinate system was a marker adhered to the dorsal landmark of wrist, with the X axis pointing radially, the Y axis pointing distally, and the Z axis pointing upwards. The coordinates of the markers measured in the global (laboratory) coordinate system (X–Y–Z) were transformed and expressed in the local coordinate system (X_0_-Y_0_-Z_0_).

Pre-amplified electromyographic (EMG) activity from the Flexor Digitorum and Extensor Digitorum locations (middle of the forearm approximately three quarters of the distance between the elbow and the wrist, ventral and dorsal side, respectively) were recorded during the performance of the tasks. Gold contact silver–chloride (AgCl) gelled surface electrodes were applied on prepared skin. EMG signals were sampled synchronously at 2,000 Hz. Subsequently, the EMG signals were band-pass filtered (zero phase shift, fourth-order Butterworth, 20 Hz low-pass, 300 Hz high-pass cut-off) to remove any interference or low frequency movement artifact and then full-wave rectified to perform magnitude analysis of EMG.

### Data analysis

To quantify the extent of coupling between muscle activation and motion, the averaged EMG and joint angles were plotted and measurements for amplitude and timing of relevant portions of the signals were analyzed. Onsets and ends of EMG bursts were identified and referred to movement onsets at the points where the signal left or returned to a baseline determined by visual inspection of the EMG and joint angles prior to movement.

**Figure 2:**
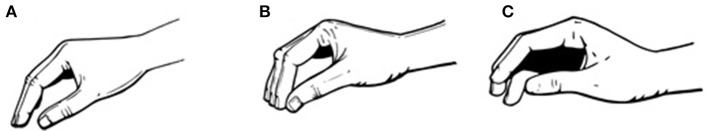
Pre-defined stages of manipulative tasks: **(A)** Formation; **(B)** manipulation; **(C)** release.

The analysis was conducted for three stages of each trial, splitting tasks into: formation of the grip, manipulation, and release; this approach increases the precision of the analysis. providing insight into the range of strategies across grasping patterns. The stages were defined by visual inspection of data and video. Purely velocity detection algorithms were not used as we wanted to observe the grasping patterns used by the participants. The formation stage was defined as the portion of the task between the start of the movement and the first contact with the object (Figure 2A). The manipulation stage was defined as the period of the task between the first contact of the dominant hand with the object and the moment no contact between the hand and the object is detected (Figure 2B). Finally, the release stage was defined as the portion of the task starting when the hand stops making contact with the object and ending with the hand back in the resting posture (Figure 2C, Gonzalez, 2016).

Muscle activation was quantified in terms of its spatiotemporal characteristics. The muscle activation patterns throughout every phase of the trials were identified by the number of bursts. Muscle activation was quantified in terms of its spatiotemporal characteristics. The muscle activation patterns throughout every phase of the trials were identified by the number of bursts. Bursts were defined based on two conditions, a peak amplitude of at least 0.01 mV and a duration of at least 0.2 s. To determine whether slowness to activate muscles interfered with performance on the tasks *maximum speed of EMG* was obtained for both finger flexor and extensor. All of these measurements were made by the same individual using consistent criteria with the timing of onsets and end of trials.

A paired samples *T*-test with Bonferroni corrections was conducted to further compare EMG bursts patterns between dexterity tests and related activities of daily living. The difference in number of bursts between flexor digitorum and extensor digitorum was computed and the means compared for each task phase across subjects. Results from the *T*-test showed no statistically significant difference between bursts patterns in most tasks (*p* > 0.05). However, difference between the manipulation phase of the VDT-Precision test and the precision daily living tasks approached significance (*p* < 0.05 and *p* < 0.02 for coin and spoon tasks respectively) as shown in Table 1.

**Table 1:** Results from paired samples test for differences in number of bursts between flexor digitorum and extensor digitorum during the formation and manipulation phases (Confidence interval at 95%).

**Extensor digitorum-flexor digitorum number of bursts difference**		**Sig. (2-tailed)**
**FORMATION PHASE**	
Pair 1	Bursts difference VDT-C-bursts difference cup	0.363
Pair 2	Bursts difference VDT-P-bursts difference spoon	0.102
Pair 3	Bursts difference PPT-bursts difference coin	0.465
Pair 4	Bursts difference VDT-P-bursts difference coin	0.175
**MANIPULATION PHASE**	
Pair 1	Bursts difference VDT-C-bursts difference cup	0.421
Pair 2	Bursts difference VDT-P-bursts difference spoon	0.013
Pair 3	Bursts difference PPT-bursts difference coin	0.576
Pair 4	Bursts difference VDT-P-bursts difference coin	0.041

*Dexterity tests and daily living tasks were paired according to the grasping pattern*.

The instantaneous flexion angles for digits 2–5 were then obtained by calculating the angle between the pre-defined segments from the local reference system. The metacarpophalangeal (MCP) flexion angle was defined as the angle between the metacarpal segment and the proximal phalanx segment. The proximal interphalangeal (PIP) flexion was calculated as the angle between the proximal phalanx segment and the middle phalangeal segment. The distal interphalangeal (DIP) flexion was defined as the angle between the middle phalangeal segment and the distal phalangeal segment. Thumb abduction/adduction was defined as the angle between the thumb's proximal phalanx segment and the segment between markers T2 and I2 (Gonzalez, 2016).

Speed of EMG was calculated as the rate of change of magnitude of EMG (the difference in EMG in consecutive samples divided by the duration of the interval). Maximum values of speed of EMG were obtained for each trial in order to determine whether slowness to activate muscles interfered with performance on the tasks.

## Results

**Figure 3:**
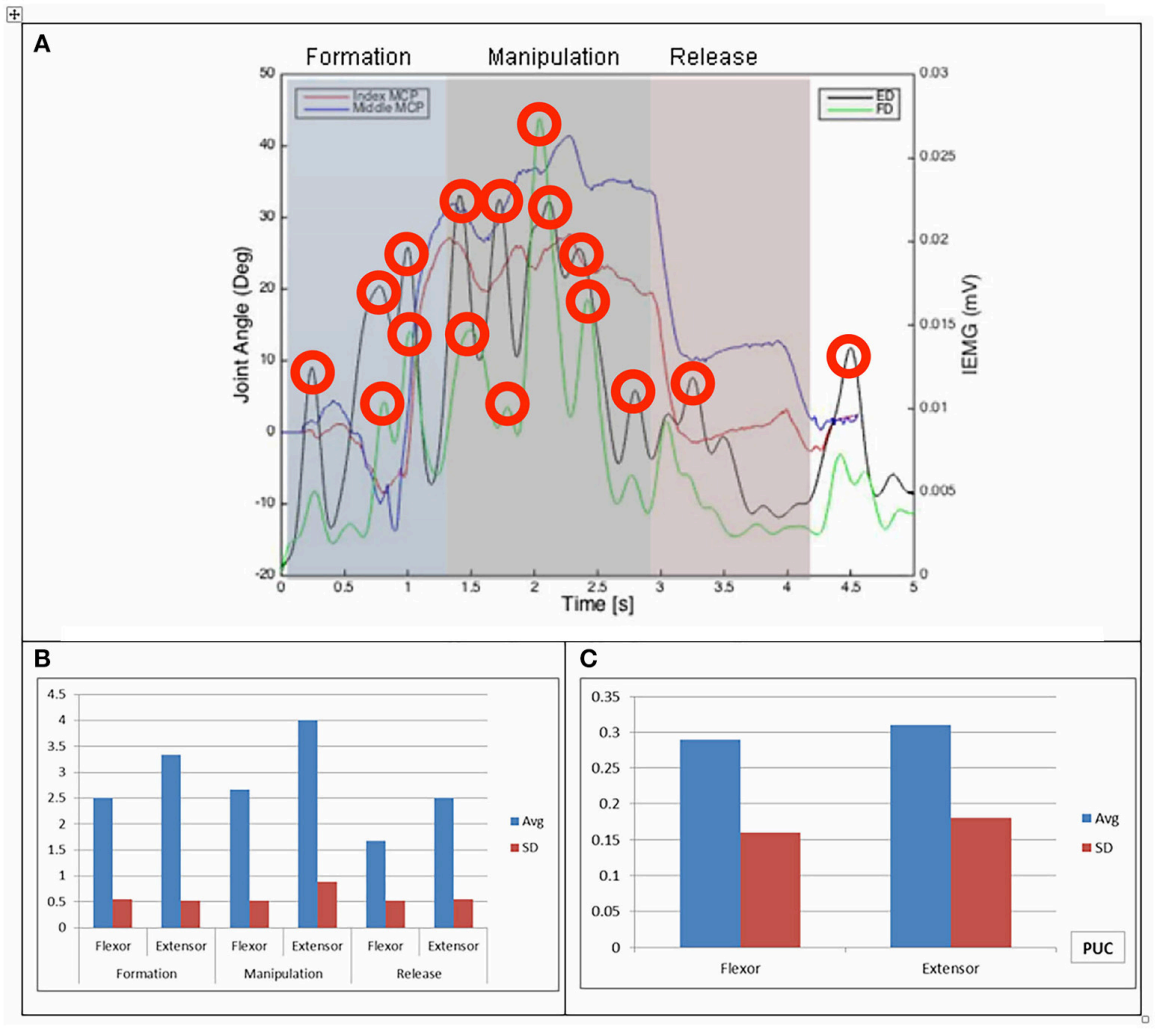
**(A)** Relationship between average IEMG and index and middle fingers flexion angles (picking up a coin). **(B)** Mean number of IEMG bursts and standard deviations (picking up a coin). **(C)** Mean peak speed of IEMG and standard deviations (picking up a coin).

**Figure 4:**
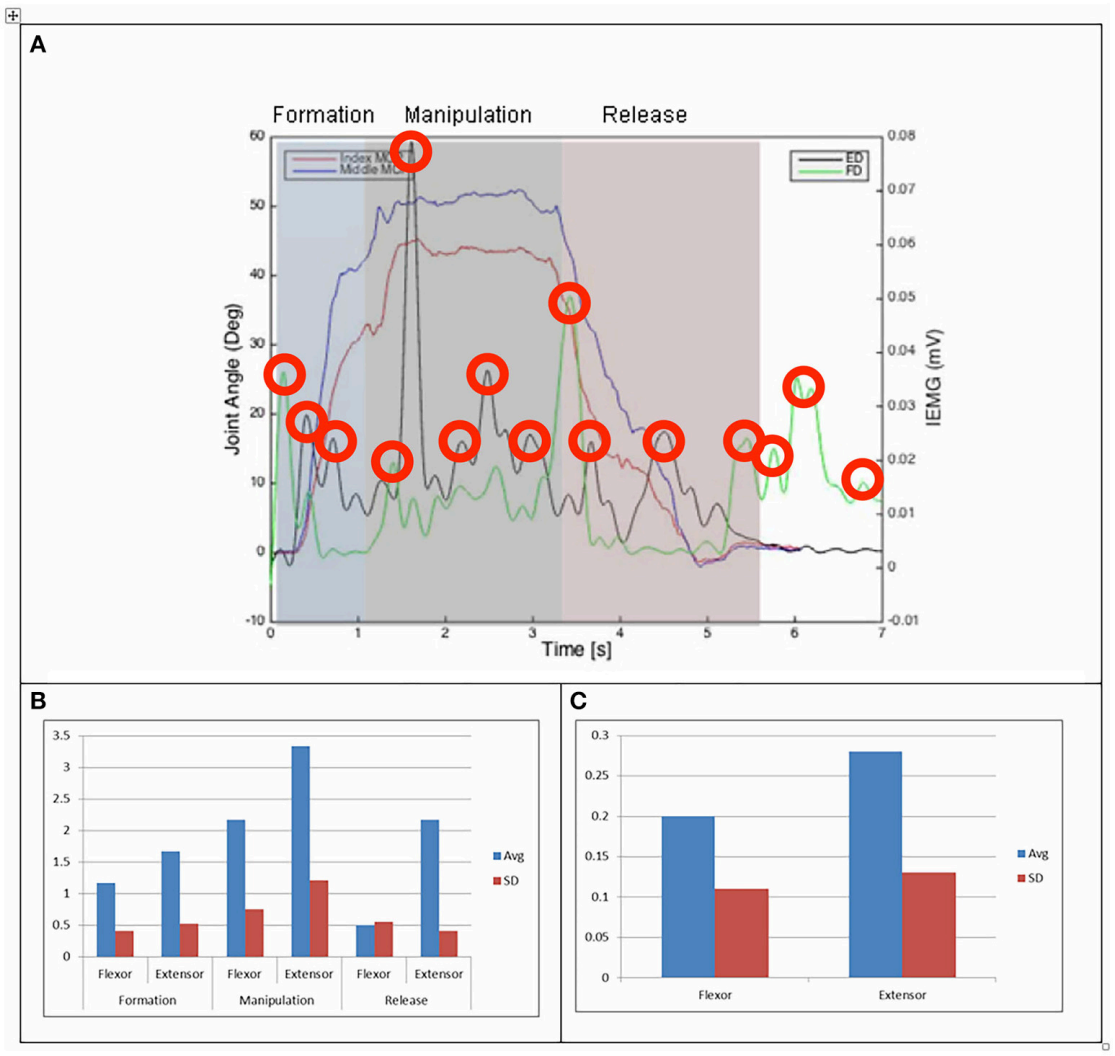
**(A)** Relationship between average IEMG and index and middle fingers flexion angles (feeding with a spoon). **(B)** Mean number of IEMG bursts and standard deviations (feeding with a spoon). **(C)** Mean peak speed of IEMG and standard deviations (feeding with a spoon).

**Figure 5:**
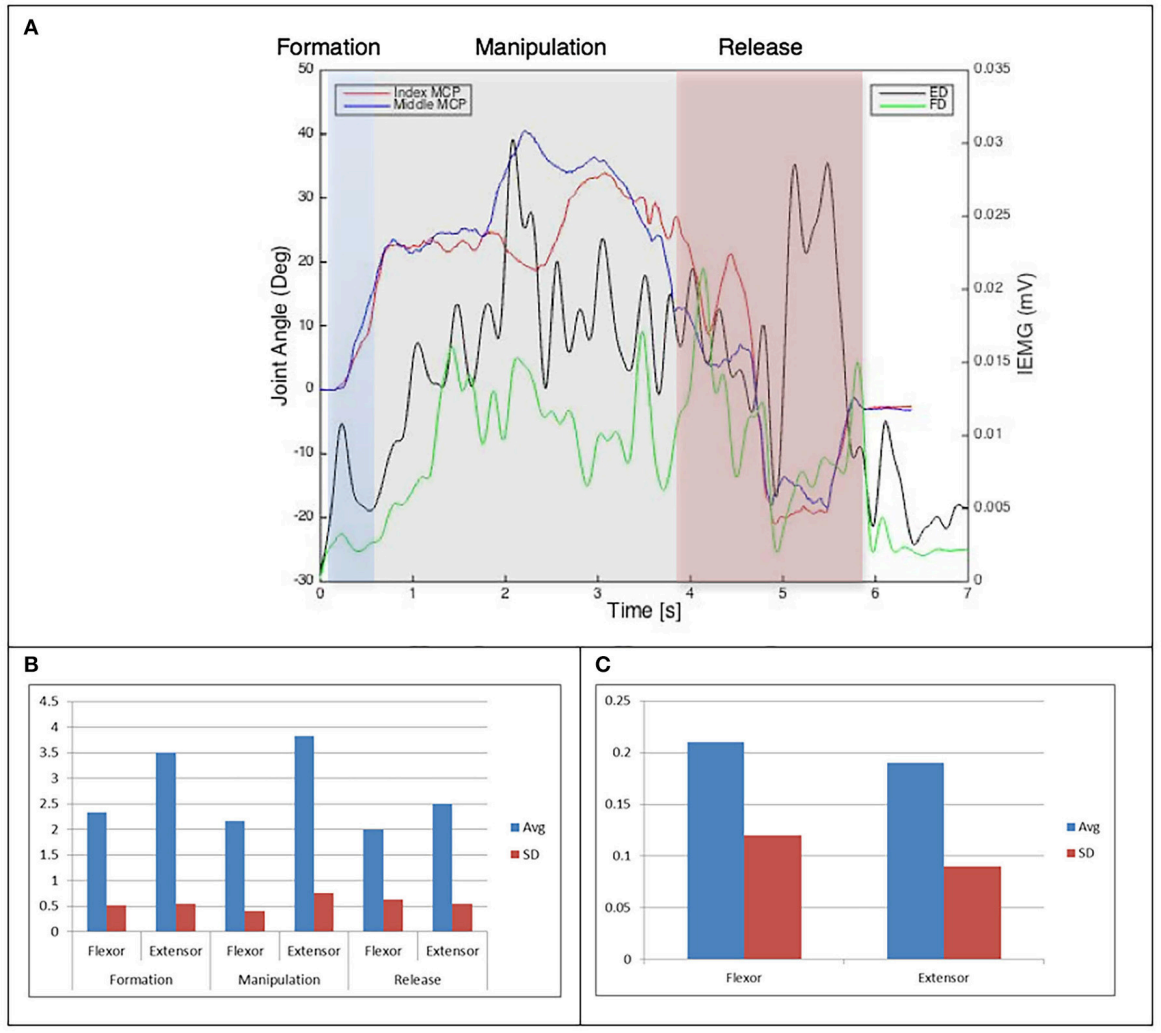
**(A)** Relationship between average IEMG and index and middle fingers flexion angles (Purdue pegboard test). **(B)** Mean number of IEMG bursts and standard deviations (Purdue pegboard test). **(C)** Mean peak speed of IEMG and standard deviations (Purdue pegboard test).

**Figure 6:**
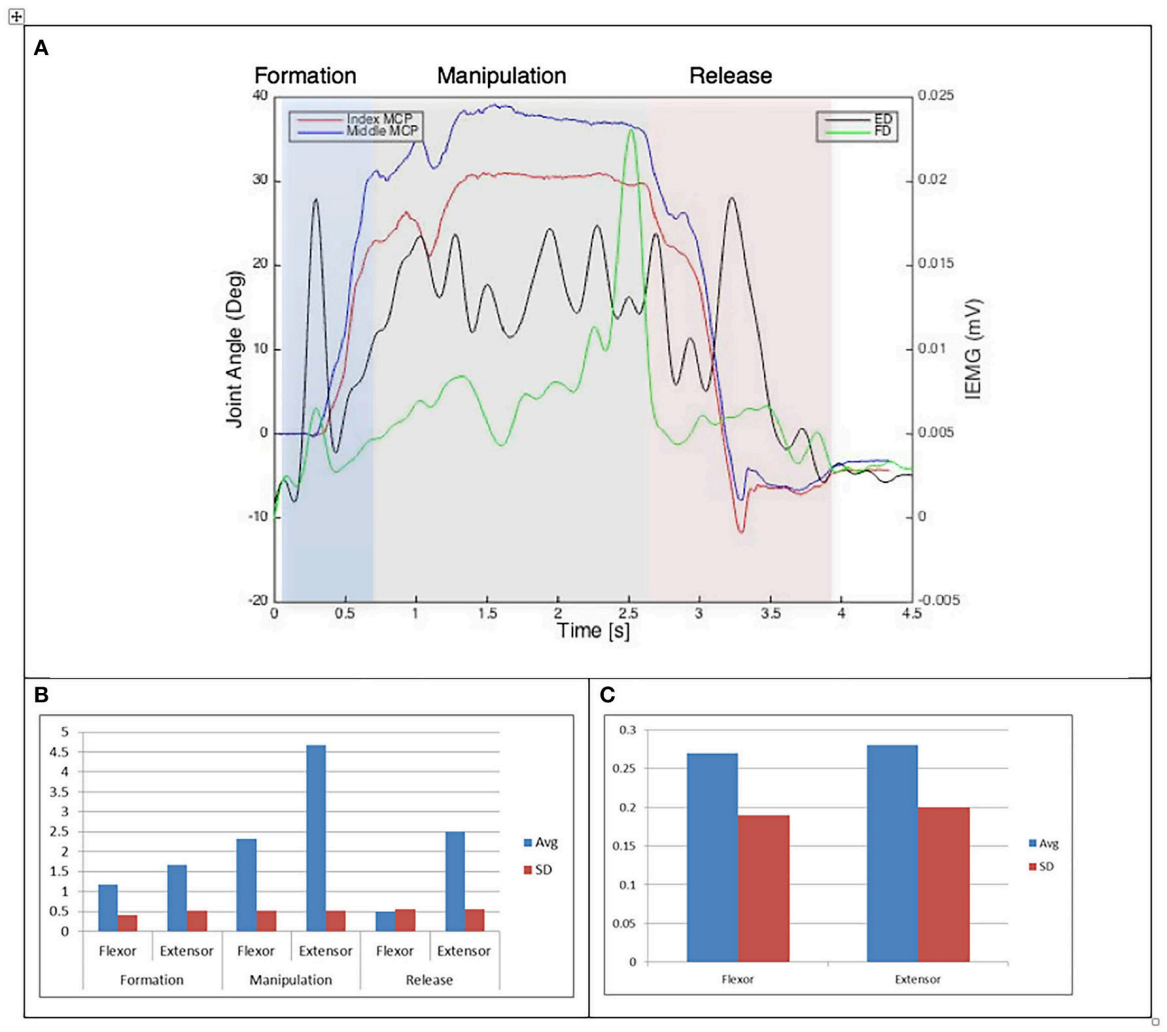
**(A)** Relationship between average IEMG and index and middle fingers flexion angles (Variable dexterity test-precision). **(B)** Mean number of IEMG bursts and standard deviations (Variable dexterity test-precision). **(C)** Mean peak speed of IEMG and standard deviations (Variable dexterity test-precision).

**Figure 7:**
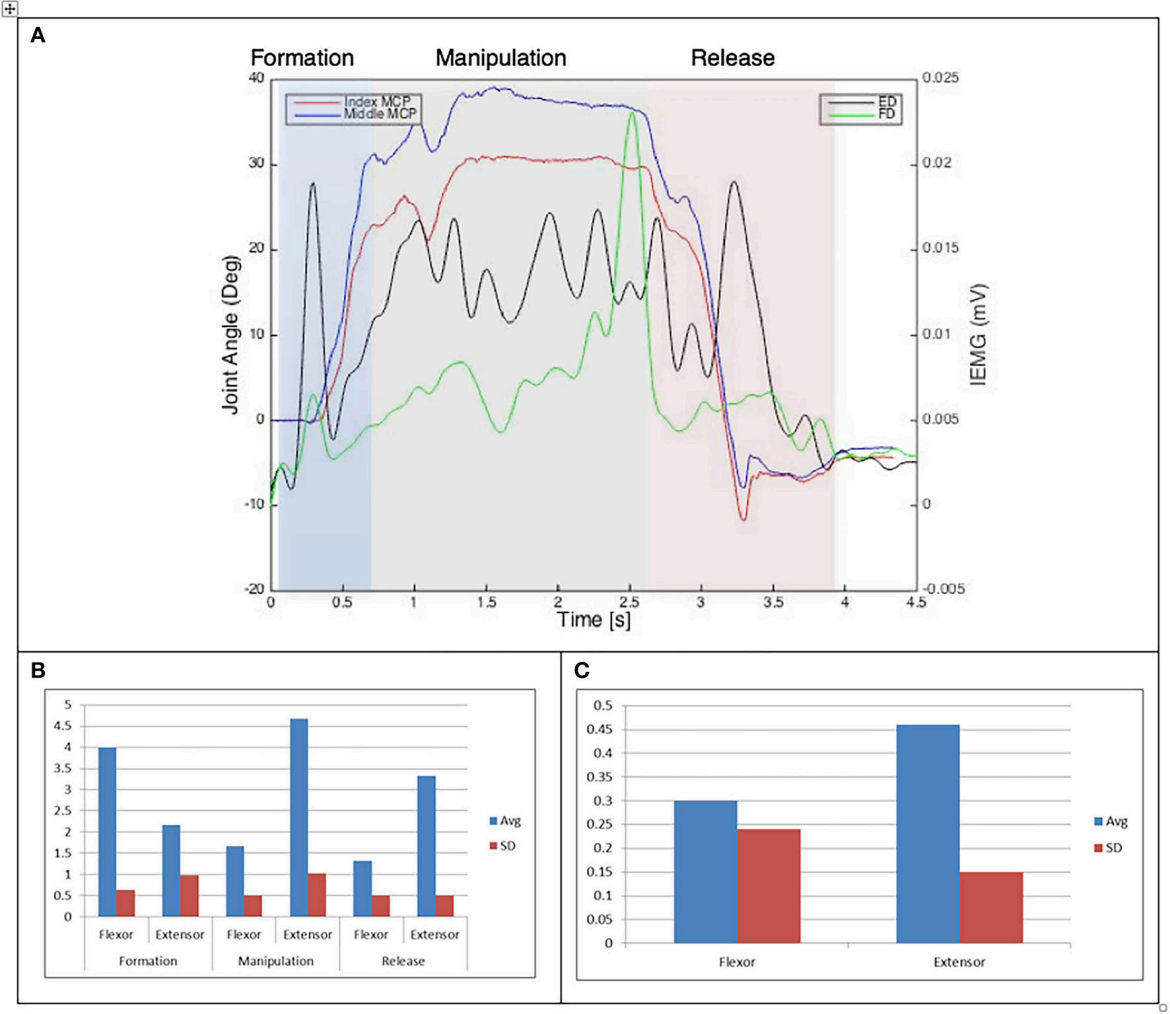
**(A)** Relationship between average IEMG and index and middle fingers flexion angles (Drinking from a cup). **(B)** Mean number of IEMG bursts and standard deviations (Drinking from a cup). **(C)** Mean peak speed of IEMG and standard deviations (Drinking from a cup).

**Figure 8:**
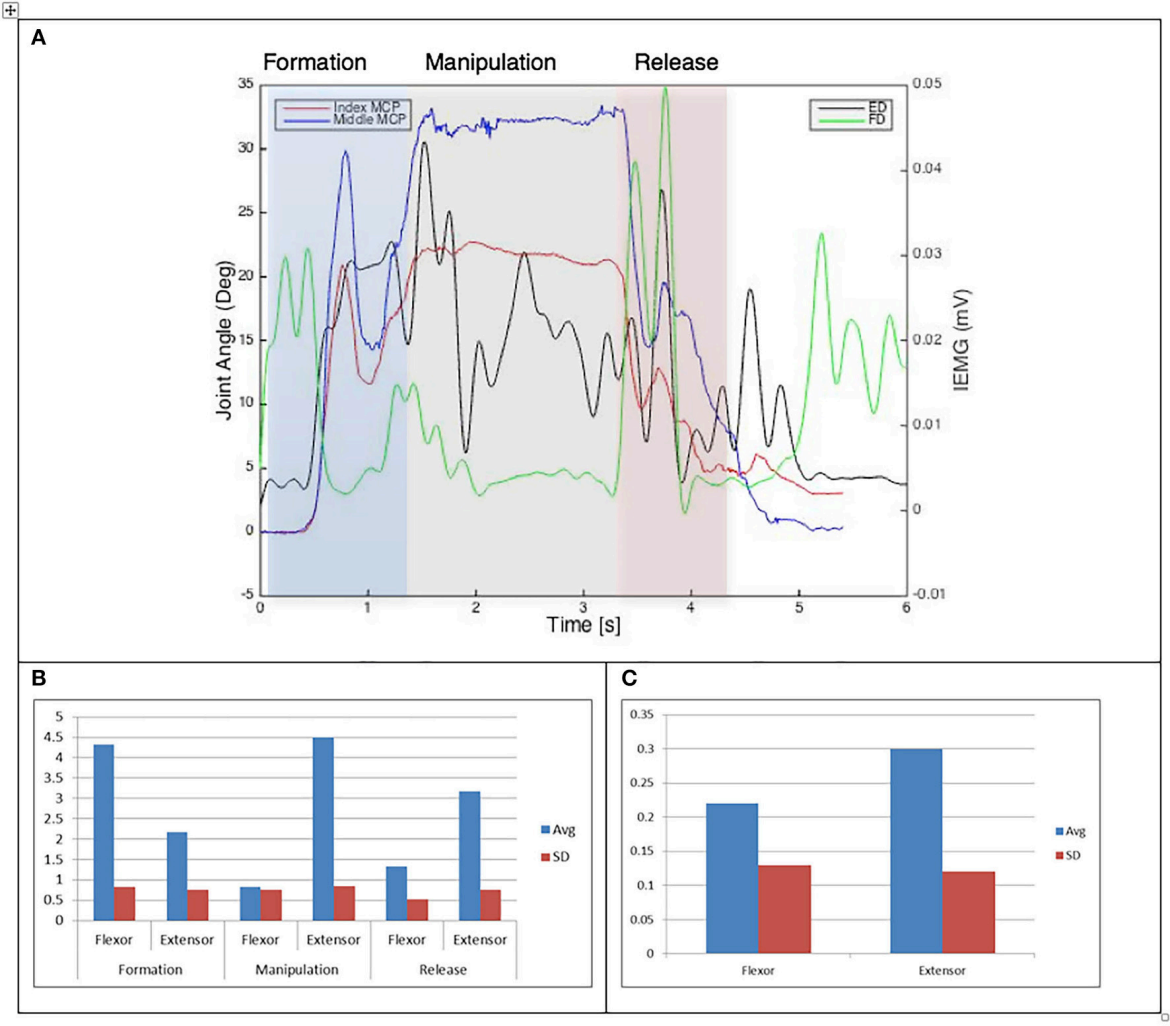
**(A)** Relationship between average IEMG and index and middle fingers flexion angles (Variable dexterity test-cylinder). **(B)** Mean number of IEMG bursts and standard deviations (Variable dexterity test-cylinder). **(C)** Mean peak speed of IEMG and standard deviations (Variable dexterity test-cylinder).

Results of the relationship of EMG patterns for the index and middle fingers, the mean number of EMG bursts and the mean peak speed of the EMG are shown in Figures 3–8 for all of the ADL and dexterity measurement tasks. Figures 3, **4** show the relationship between average EMG and index and middle finger flexion angles for feeding with a spoon and picking up a coin with EMG bursts complying where our pre-defined conditions for a “burst” (as described earlier) are shown circled.

The drinking from a cup task shows higher speed of bursts from both flexor and extensor muscles than those required by the dexterity tests, with the peak mean speed of 0.46 (mV/s) being over twice that of the Purdue Pegboard test with a peak mean speed of 0.19 for the flexor digitorum. The peak mean speed was seen to be generally higher for the extensor digitorum in all cases except the Purdue Pegboard where the flexor digitorum was seen to be slightly higher at 0.21. Picking up a coin, the Variable Dexterity test-Cylinder, the Variable Dexterity test-Precision and feeding with a spoon tasks all produced similar peak mean speeds for the extensor digitorum. More variation for these tasks was seen for the flexor digitorum with range of 0.29 for picking up a coin to 0.2 for feeding with a spoon.

Examining the results across the whole task from formation, manipulation and release significant differences can be seen. Picking up a coin and the Purdue Pegboard Test show similar patterns with a higher number of EMG bursts for the extensor and flexor digitorum for the formation and release phases, whilst the peak mean speed value occurring on the extensor digitorum during the manipulation phase. Similarly the feeding with a spoon shows a similar pattern variable dexterity test-precision and the drinking from a cup shows a similar pattern to the variable dexterity test-precision.

In general, no statistically significant differences were found between tasks and dexterity test performance for most comparisons, with differences between the spoon task and the VDT-Precision test approaching significance after Bonferroni correction (Table 1). A Shapiro-Wilk test was used to test for normality with *p* > 0.05 indicating normal distribution of data.

## Discussion

The aim of thisstudy was to characterize the muscle activation patterns which underlie the performance of two commonly used grasping patterns and compare the characteristics of such patterns during dexterity tests and activities of daily living. This study has shown that, although minor differences are observable in both kinematic and muscle activation patterns, under these experimental conditions, the selected dexterity tests effectively reflect muscle activation patterns observed during the performance of tasks related to activities of daily living.

The size of the object size was observed to be particularly significant in the precision grip tasks, with two identifiable movement and EMG patterns relative to the size of the object. Movement and activation patterns observed during cylinder grip tasks were generally consistent for the VDT and the drinking task.

The grip formation phase from tasks requiring the precision grasping pattern had consistently comparable patterns of bursts from the extensor and flexor muscles for all tasks and subjects. This could be due to adequate finger coordination during the approach and formation phases before contact with the object was made. During the manipulation phase of the precision tasks activity from the extensor digitorum had a significantly larger number of bursts when compared to activity from the flexor digitorum. This behavior may be due to subjects struggling to maintain control of the relatively small objects used for these tasks. These manipulation patterns were particularly evident during performance of small object task (picking up a coin and Purdue Pegboard Test). Metacarpophalangeal joint angles from index and middle fingers during the Purdue Pegboard and the coin task showed further irregularity that was reflected as muscle activity patterns with larger presence of bursts from both flexor and extensor muscles.

During the release phase of precision tasks activity from the extensor digitorum was consistently larger in magnitude and number of bursts across subjects and tasks. This behavior was expected as it was during this phase that subjects started extending the fingers freely to release the object and return the hand to the resting position.

The grip formation phase of cylinder tasks was characterized by a greater number of bursts from the flexor digitorum when compared to the extensor digitorum. This behavior was consistent with information obtained from finger kinematics, as joint flexion angles were increasing with the hand preparing to make contact and manipulate the cylindrical objects. During the manipulation phase of cylinder tasks, activity from the flexor digitorum decreased considerably with the extensor digitorum generating consistently more bursts. This pattern was attributed to the grasping pattern being formed and the subject interacting the object struggling to maintain control during the manipulation. Flexion angles from the metacarpophalangeal joints were consistently regular during the manipulation phase, with little or no sudden changes indicating subjects did not struggle to perform the tasks. Activity from the extensor digitorum was significantly higher during the release phase of cylinder tasks when compared to activity from the flexor digitorum, as fingers were extending to release the object and the hand was returning to the resting position on the table.

Generally, muscle activity was larger from cylinder tasks when compared to precision tasks. This behavior can be attributed to the larger and heavier objects used for cylinder tasks. Although object size was also reflected on joint flexion angles being considerably higher during precision tasks, it can be observed the effect of object weight was larger than amplitude of movement (joint flexion) when comparing muscle activity.

Results from the paired samples *T*-test showed no statistically significant difference in number of bursts difference between most grip-related dexterity tests and daily living tasks. However, differences approached statistical significance when comparing the VDT-Precision with the precision tasks during the manipulation phase. This difference in activation patterns may be due to object size difference between precision tasks and the VDT-Precision.

It has been demonstrated by Canning et al. (2000) and Fellows et al. (1994) that there is a relation between dexterity and muscle activation patterns, with high dexterity being generally related to minimal muscle activity during task performance, while excessive muscle activation is generally observed in low dexterity patients when no load is applied.

The minor differences observed in EMG activity between flexor and extensor muscles in the performance of activities of daily living and dexterity tests may be due to a change in the manipulation strategies depending on the size of the object and the familiarity of participants with the activities of daily living, in contrast with their knowledge of the dexterity tests.

Maximum speed of EMG, used as a measure of speed to activate the muscles under analysis, provided further evidence of these differences between tasks, with higher values generally observed from activities of daily living. This patterns of rapid muscle activation may be related to the higher levels of dexterity of subjects when performing familiar tasks.

## Conclusion

The work has shown that a common rehabilitation test such as the Purdue Pegboard test is a poor representation of the muscle activation patterns for activities of daily living. EMG and joint angle patterns from the Variable Dexterity Tests which has been designed to more accurately reflect a range of ADl's, were consistently comparable with tasks requiring precision and cylinder grip, reaffirming the importance of object size and shape when attempting to accurately assess hand function. The muscle activation patterns identified in this study reflect the differences in muscle activation when generating a range of grasping patterns which conform with daily living demands which had not previously be identified. Accordingly, the correct assessment of dexterity is a fundamental objective for rehabilitation efforts. These results would suggest that during assessment and rehabilitation, a wide range of flexible inclusive tests and tasks be provided to inclusively evaluate hand functionality throughout the large spectrum of daily living activities that make up a person's independent life.

## Future work

The main focus for future work will be on the limitations of this research. A larger sample size will provide information of the validity and reliability of the analysis techniques, while at the same time allowing a robust study of the accuracy and repeatability of the data acquisition protocols.

A number of alternative approaches to the measurement of human movement variability and pattern recognition were not explored (velocity analysis, vector coding, factor analysis, dynamic stability methods), and their viability and accuracy has to be assessed and compared with the techniques proposed in this work.

In addition, although the Variable Dexterity Test proved to be a flexible and cost-effective experimental tool, it has yet to be fully developed in order to be reliably used as dexterity assessment method for clinical practice (Gonzalez, 2016).

## Author contributions

JR was leading the work at Sheffield University, working closely with VG to carry out the work, defining both the experimental tests and collaborating with the analysis of the results. AY contributed to the analysis and application of the work and the paper was co-written by all the authors.

### Conflict of interest statement

The authors declare that the research was conducted in the absence of any commercial or financial relationships that could be construed as a potential conflict of interest.

## References

[B1] Aaron D. H. Jansen C. W. (1992). Development of the functional dexterity test (FDT): construction, validity, reliability, and normative data. J. Hand Ther. 16, 12–21. 12611441 10.1016/s0894-1130(03)80019-4

[B2] Barbič J. Safonova A. Pan J.-Y. Faloutsos C. Hodgins J. K. Pollard N. S. (2004). Segmenting Motion Capture Data Into Distinct Behaviors. London, ON; Ontario: Canadian Information Processing Society.

[B3] Bodenheimer B. Rose C. Rosenthal S. (1997). Introduction 2 basic motion capture process, in The Process of Motion Capture : Dealing With the Data, 1–14.

[B4] Berger D. J. d'Avella A. (2014). Effective force control by muscle synergies. Front. Comput. Neurosci. 8:46. 10.3389/fncom.2014.00046 24860489 PMC4029017

[B5] Braido P. Zhang X. (2004). Quantitative analysis of finger motion coordination in hand manipulative and gestic acts. Hum. Mov. Sci. 22, 661–678. 10.1016/j.humov.2003.10.001 15063047

[B6] Canning C. G. Ada L. O'Dwyer N. J. (2000). Abnormal muscle activation characteristics associated with loss of dexterity after stroke. J. Neurol. Sci. 176, 45–56. 10.1016/S0022-510X(00)00305-1 10865092

[B7] Cappozzo A. Catani F. Croce U. D. Leardini A. (1995). Position and orientation in space of bones during movement: anatomical frame definition and determination. Clin. Biomech. 10, 171–178. 10.1016/0268-0033(95)91394-T 11415549

[B8] Cappozzo A. Della Croce U. Leardini A. Chiari L. (2005). Human movement analysis using stereophotogrammetry. Part 1: theoretical background. Gait Post. 21, 186–196. 10.1016/j.gaitpost.2004.01.010 15639398

[B9] Carpinella I. Mazzoleni P. Rabuffetti M. Thorsen R. Ferrarin M. (2006). Experimental protocol for the kinematic analysis of the hand: definition and repeatability. Gait Posture 23, 445–454. 10.1016/j.gaitpost.2005.05.001 15978812

[B10] Castellini C. Van Der Smagt P. (2013). Evidence of muscle synergies during human grasping. Biol. Cybernet. 107, 233–245. 10.1007/s00422-013-0548-4 23370962

[B11] Chiari L. Della Croce U. Leardini A. Cappozzo A. (2005). Human movement analysis using stereophotogrammetry. Part 2: instrumental errors. Gait Posture 21, 197–211. 10.1016/j.gaitpost.2004.04.004 15639399

[B12] Degeorges R. Parasie J. Mitton D. Imbert N. Goubier J. N. Lavaste F.. (2005). Three-dimensional rotations of human three-joint fingers: an optoelectronic measurement. Preliminary results. Surg. Radiol. Anat. 27, 43–50. 10.1007/s00276-004-0277-4 15316760

[B13] Dipietro L. Sabatini A. M. Dario P. (2008). A survey of glove-based systems and their applications. IEEE Trans. Syst. Cybern. 38, 461–482. 10.1109/TSMCC.2008.923862

[B14] Duque J. Masset D. Malchaire J. (1995). Evaluation of handgrip force from EMG measurements. Appl. Ergon. 26, 3–4. 10.1016/0003-6870(94)00003-H 15677002

[B15] Ellis B. Bruton A. (2002). A study to compare the reliability of composite finger flexion with goniometry for measurement of range of motion in the hand. Clin. Rehabil. 16, 562–570. 10.1191/0269215502cr513oa 12194627

[B16] Engel K. C. Flanders M. Soechting J. F. (1997). Anticipatory and sequential motor control in piano playing. Exp. Brain Res. 113, 189–199. 10.1007/BF02450317 9063705

[B17] Fellows S. J. Kaus C. Thilmann A. F. (1994). Voluntary movement at the elbow in spastic hemiparesis. Ann. Neurol. 36, 397–407. 10.1002/ana.410360311 8080247

[B18] Fleishman E. A. Hempel W. E. (1954). A factor analysis of dexterity tests. Pers. Psychol. 7, 15–32. 10.1111/j.1744-6570.1954.tb02254.x

[B19] Gonzalez V. (2016). Development of a Dexterity Assessment Method, PhD thesis, University of Sheffield, Available online at http://etheses.whiterose.ac.uk/15609/

[B20] Gonzalez V. Rowson J. Yoxall A. (2015). Development of the variable dexterity test: construction, reliability and validity. Int. J. Ther. Rehabil. 22, 174–180. 10.12968/ijtr.2015.22.4.174

[B21] Häger-Ross C. Schieber M. H. (2000). Quantifying the independence of human finger movements: comparisons of digits, hands, and movement frequencies. J. Neurosci. 20, 8542–8550. 10.1523/JNEUROSCI.20-22-08542.2000 11069962 PMC6773164

[B22] Huang H. Wolf S. L. He J. (2006). Recent developments in biofeedback for neuromotor rehabilitation. J. Neuroeng. Rehabil. 3:11. 10.1186/1743-0003-3-11 16790060 PMC1550406

[B23] Landsmeer J. M. (1962). Power grip and precision handling. Ann. Rheum. Dis. 21, 164–170. 10.1136/ard.21.2.164 14461996 PMC1007266

[B24] Latash M. L. Scholz J. F. Danion F. Schöner G. (2002). Finger coordination during discrete and oscillatory force production tasks. Exp. Brain Research 146, 419–432. 10.1007/s00221-002-1196-4 12355270

[B25] Liarokapis M. V. Artemiadis P. K. Katsiaris P. T. Kyriakopoulos K. J. Manolakos E. S. (2012). Learning human reach-to-grasp strategies: towards EMG-based control of robotic arm-hand systems, in 2012 IEEE International Conference on Robotics and Automation (Guangzhou), 2287–2292.

[B26] Light C. M. Chappell P. H. Kyberd P. J. (2002). Establishing a standardized clinical assessment tool of pathologic and prosthetic hand function: normative data, reliability, and validity. Arch. Phys. Med. Rehabil. 83, 776–783. 10.1053/apmr.2002.32737 12048655

[B27] Maier M. A. Hepp-Reymond M. C. (1995). EMG activation patterns during force production in precision grip. II. Muscular synergies in the spatial and temporal domain. Exp. Brain Res. 103, 123–136. 10.1007/BF00241970 7615028

[B28] Metcalf C. D. Notley S. V. Chappell P. H. Burridge J. H. Yule V. T. (2008). Validation and application of a computational model for wrist and hand movements using surface markers. IEEE Trans. Biomed. Eng. 55, 1199–1210. 10.1109/TBME.2007.908087 18334414

[B29] Moeslund T. B. Granum E. (2001). A survey of computer vision-based human motion capture. Comput. Vis. Image Underst., 81, 231–268. 10.1006/cviu.2000.0897

[B30] NAPIER J. R. (1956). The Prehensile movements of the human hand. J. Bone Joint Surg. 38-B, 902–913. 10.1302/0301-620X.38B4.902 13376678

[B31] Osu R. Ota K. Fujiwara T. Otaka Y. Kawato M. Liu M. (2011). Quantifying the quality of hand movement in stroke patients through three-dimensional curvature. J. Neuroeng. Rehabil. 8:62. 10.1186/1743-0003-8-62 22040326 PMC3377918

[B32] Peterson L. R. Centre R. (1999). The moberg pickup test. J. Hand Ther. 12, 309–312. 10622197 10.1016/s0894-1130(99)80069-6

[B33] Rash G. S. Belliappa P. P. Wachowiak M. P. Somia N. N. Gupta A. (1999). A demonstration of validity of 3-D video motion analysis method for measuring finger flexion and extension. J. Biomech. 32, 1337–1341. 10.1016/S0021-9290(99)00140-2 10569712

[B34] Sancho-Bru J. L. Jarque-Bou N. J. Vergara M. Pérez-González A. (2014). Validity of a simple videogrammetric method to measure the movement of all hand segments for clinical purposes. Proc. Inst. Mech. Eng. H. J. Eng. Med. 228, 182–189. 10.1177/0954411914522023 24503512

[B35] Small C. F. Bryant J. T. Pichora D. R. (1992). Rationalization of kinematic descriptors for three-dimensional hand and finger motion. J. Biomed. Eng. 14, 133–141. 10.1016/0141-5425(92)90018-G 1564920

[B36] Soechting J. F. Flanders M. (1997). Flexibility and repeatability of finger movements during typing: analysis of multiple degrees of freedom. J. Comput. Neurosci. 4, 29–46. 10.1023/A:1008812426305 9046450

[B37] Surrey L. R. Nelson K. Delelio C. Mathie-Majors D. Omel-Edwards N. Shumaker J.. (2003). A comparison of performance outcomes between the Minnesota rate of manipulation test and the minnesota manual dexterity test. Work, 20, 97–102. 12671203

[B38] TAYLOR C. L. SCHWARZ R. J. (1955). The anatomy and mechanics of the human hand. Artif. Limbs 2, 22–35. 13249858

[B39] Thakur P. H. Bastian A. J. Hsiao S. (2008). Multidigit movement synergies of the human hand in an unconstrained haptic exploration task. J. Neurosci. 28, 1271–1281. 10.1523/JNEUROSCI.4512-07.2008 18256247 PMC6671569

[B40] Tiffin J. Asher E. (1948). The Purdue pegboard test; norms and studies of reliability and validity. J. Appl. Psychol. 32, 234–247. 10.1037/h0061266 18867059

[B41] van den Doel K. Ascher U. M. Curt A. Steeves J. Pai D. K. (2008). Computed myography (CMG): three dimensional reconstruction of motor functions from surface EMG data, in Annual International Conference of the IEEE Engineering in Medicine and Biology Society 2008 (Vancouver, BC: IEEE Engineering in Medicine and Biology Society), 550–554. 10.1109/IEMBS.2008.4649212 19162715

[B42] Warlow O. M. Lawson S. E. (2012). A technique for motion capture of the finger using functional joint centres and the effect of calibration range of motion on its accuracy. Proc. Inst. Mech. Eng. H J. Eng. Med. 226, 360–367. 10.1177/0954411912442133 22720388

[B43] Winges S. A. Weber D. J. Santello M. (2003). The role of vision on hand preshaping during reach to grasp. Exp. Brain Res. 152, 489–498. 10.1007/s00221-003-1571-9 12898088

